# New Insights into Breast and Endometrial Cancers

**DOI:** 10.3390/cancers12092595

**Published:** 2020-09-11

**Authors:** Yasuhiro Miki

**Affiliations:** Department of Disaster Obstetrics and Gynecology, International Research Institute of Disaster Science (IRIDeS), Tohoku University, Sendai 980-0000, Japan; miki@patholo2.med.tohoku.ac.jp; Tel.: +81-22-273-6284; Fax: +81-22-273-6284

**Keywords:** breast cancer, endometrial cancer, therapeutic target molecule, prognostic factor, hormone

## 1. Preface

**Figure 1:**
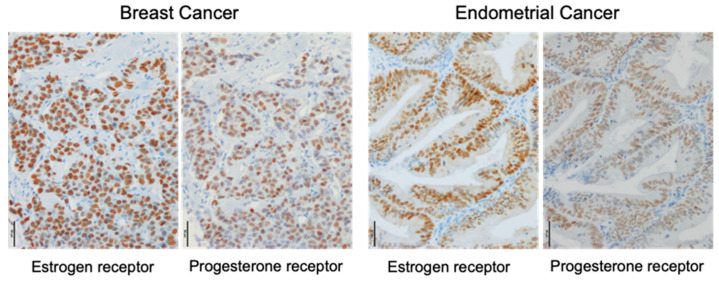
Immunohistochemistry of estrogen and progesterone receptors in breast and endometrial cancers. Immunoreactivities of the estrogen receptor α and progesterone receptor were detected in the nucleus (brown) of carcinoma cells. Scale bar, 50 μm.

Cancers of the breast and endometrium are some of the most common cancers affecting women. These cancers show estrogen-dependent proliferation in several cases; therefore, hormone therapeutic strategies are employed. Tamoxifen is an effective hormone therapy that is traditionally used for estrogen receptor-positive breast cancer patients. However, in endometrial cancer, tamoxifen treatment increases the risk of a poor prognosis type of endometrial cancer [1]. The therapeutic strategy for breast cancer is defined based on the immunohistochemical expression of biomarkers, mainly the estrogen receptor (ER), progesterone receptor (PgR), and Human Epidermal Growth Factor Receptor 2 (HER2). However, a type of breast cancer, which is negative for all these biomarkers—the so-called triple-negative breast cancer (TNBC)—is more aggressive; hence, new therapeutic strategies are needed. Endometrial cancer is classified into type I and type II based on the histologic type, grade, and gene expression pattern. Type I endometrial cancer is typically G1 and G2 endometrioid cancer, which is thought to be caused by excess estrogen [2]. Type II endometrial cancer includes papillary serous carcinoma, clear cell carcinoma, carcinosarcoma, and G3 cancer [2]. Unlike type I endometrial cancer, type II cancer is not caused by excess estrogen [2]. Therefore, in type II endometrial cancer, the receptors for estrogen and progesterone are not identified or are at low levels in tumor tissues. In particular, clear cell carcinoma does not have both ER and PgR [2]. The immunohistochemistry results of ER and PgR in breast and endometrial cancers are shown in Figure 1.

An anti-estrogen therapy is typically used in ER-positive breast cancer; however, its effect is believed to be limited in type I endometrial cancer. Both breast and endometrial cancers are categorized as hormone-dependent cancers; however, the differences and similarities of estrogen signals in each cancer are unclear. Furthermore, it may be useful to better understand breast and endometrial cancers by comparing their hormone-dependent and hormone-independent properties. The widespread use of genomic analysis has led to further progress in cancer classification. For instance, integrated genomic characterization of endometrial cancer by The Cancer Genome Atlas (TCGA) consortium classified endometrial cancer into four subgroups based on prognosis as follows: POLE/ultra-mutated, microsatellite instable/hyper-mutated, copy number low/TP53 wild type, and copy-number high/TP53 mutant [3,4]. The evolution of analysis technology is expected to deepen further research on patients with both breast and endometrial cancers. In this Special Issue—New Insights into Breast and Endometrial Cancer—the latest information from 10 original articles and one review article on breast cancer, and five original and four review articles on endometrial cancer provided by international leaders, has been compiled.

## 2. Breast Cancer

The biomarkers that showed potential for therapeutic targets have been reported and potential predicted biomarkers for resistance to existing therapies have been examined. Both natural and synthetic estrogens, which have an agonistic effect on ERα, promoted the phosphorylation of heat shock factor 1 (HSF1) in the ER-positive breast cancer cell line [5]. In ER-positive breast cancer patients, a high level of HSF1 was reported to be correlated with a worse prognosis [5]. In a mouse model transplanted with tamoxifen-resistant breast cancer cells, double inhibition of ER and nuclear transport protein exportin 1, caused a metabolic shift and autophagy, resulting in prolonged tumor regression [6]. It has been observed that cyclin-dependent kinase (CDK)4/6 inhibitors, such as palbociclib, have recently been playing an important role in the treatment of patients with ER-positive breast cancer. However, palbociclib-resistant breast cancer has already been reported. In an in vitro study, the combined use of the novel virotherapy oncolytic adenovirus (OAd) was effective in palbociclib-resistant ER-positive breast cancer [7]. A combination therapy of OAd and CDK4/6 inhibitors is expected to be applied clinically. Prognostic analysis has also been examined using circulating tumor cells (CTC) and stromal cells in the cancer microenvironment [8,9]. Several genes associated with prognosis have been extracted from a group of genes that are upregulated in the mesenchymal CTC, supporting the usefulness of the less invasive CTC examination [8]. Several studies have focused on stromal cells, particularly cancer-associated fibroblasts (CAF), in the cancer microenvironment. Phosphodiesterase 5/C-X-C motif chemokine 16 signaling occurring in CAF has been reported to be involved in poor patient prognosis [9]. These studies show the usefulness of focusing not only on the parenchymal cells but also on the cells in breast cancer. A previous study has suggested that in addition to the importance of CAF, interaction with adipocytes plays a major role in the breast cancer microenvironment [10]. In the study, the role of micoRNAs in the interaction between cancer cells and adipocytes was explained [10]. HER2 gene (ERBB2) amplification and overexpression are found in a variety of human cancers including breast cancer. In a previous study, a high frequency of ERBB2-activating mutations in an invasive lobular breast carcinoma with pleomorphic features was observed [11]. Typical invasive lobular carcinoma has a good prognosis; however, pleomorphic invasive lobular carcinoma exhibits a low hormone dependence and high proliferative potential. A clinical study on HER2 inhibitory therapy for breast cancer patients with pleomorphic invasive lobular carcinoma is needed. ***TNBC***: Regarding TNBC, notable therapeutic findings and its target factor has been reported. When calcitriol (1,25-dihydroxyvitamin D3) was administered in combination with phytoestrogen curcumin or resveratrol, the anti-angiogenic effect exerted by calcitriol alone was enhanced [12]. In a TNBC cell line, the intracellular influx of Ca^2+^ through Canonical transient receptor potential isoform 3 (TRPC3) was considered to maintain the expression of Ras GTPase-activating protein 4 (RASA4), which inhibits the Ras-Mitogen-activated protein kinase (MAPK) pathway, leading to the proliferation and resistance to apoptosis [13]. TNBC is the most immunogenic breast cancer subtype; an increasing number of PD-L1 expression levels and immune checkpoint inhibitors have been emerging as potential therapeutic options for TNBC. A previous study has provided information on the prognosis of tumor-infiltrating lymphocytes (TILs), androgen receptor (AR), and forkhead box A1 (FOXA1) in TNBC [14]. In the study, patients with low TIL scores exhibited low survival and the combination of TIL levels and AR or FOXA1 expression affected the clinical outcome [14]. In another study, a gene expression profiling meta-analysis revealed novel gene signatures related tyrosine kinase in TNBC [15]. Therapeutic target molecules are urgently needed for the treatment of TNBC, for which a comprehensive gene expression analysis of candidate factors by meta-analysis is considered a useful tool.

## 3. Endometrial Cancer

Regarding endometrial cancer, the latest research information on therapeutic target molecules, predictive markers of therapeutic effect, and prognostic factors has been collected. The levonorgestrel-releasing intrauterine system (LNG-IUS) has been reported to reduce the growth of atypical endometrial hyperplasia and early endometrioid endometrial adenocarcinomas. However, there is no marker for selecting the patients who would benefit from the LNG-IUS therapy. In a previous study, the relationship between serum or intratumoral human epididymis protein 4 (HE4) and the effect of LNG-IUS was examined [16]. Serum and intratumoral HE4 is detected at high levels in aggressive endometrial cancers. Therefore, serum HE4 has been considered a biomarker for predicting the LNG-IUS response [16]. Recently, attention has focused on the crucial role of the DNA damage response (DDR) in the mechanism of chemoresistance in various types of cancer. The cross-talking ataxia telangiectasia mutated and Rad3 related and checkpoint kinase 1 (ATR) and ataxia telangiectasia mutated and Rad3 related and checkpoint kinase 2 (ATM) pathways are recognized as the main pathways of DDR. In vitro studies of endometrial cancer showed that the cytotoxic effects of chemo-drugs and irradiation were enhanced by the co-treatment of both ATR and ATM inhibitors, respectively [17]. In the exploration for biomarkers, a cluster of microRNAs specifically expressed in endometrial cancer patients was identified by a unique method. A novel approach was provided to identify highly sensitive and specific biomarkers of endometrial cancer using extracellular vesicles isolated from the peritoneal lavage of patients [18]. Non-coding RNA that does not code for proteins was considered to be noise associated with transcription. However, the regulatory effect of gene expression by short non-coding RNAs, the so-called RNA interference, has been clarified, and the importance of non-coding RNAs such as microRNA and long non-coding RNA has been widely recognized. Two excellent review articles on the significance of non-coding RNA in endometrial cancer have been produced, and microRNA and long non-coding RNA in endometrial cancer are expected as therapeutic targets, prognostic markers, and markers for predicting drug effects or resistance [19,20]. Further bioinformatic analysis has been performed for comprehensive gene expression analysis. HiChIP chromatin loop analysis of an endometrial cell line was performed to identify the candidate target genes at 16 known endometrial cancer GWAS risk loci [21]. These data are expected to provide a very useful resource for genetic information on the risk of endometrial cancer and genetic studies of other diseases associated with the endometrium. Several biomarkers in the blood and tissues for endometrial cancer diagnosis have been reported, but none have been translated into clinical use [22]. High-throughput proteomics [22] and advanced genomics are considered useful tools to find biomarkers for early detection of endometrial cancer. It is desirable that these examinations be performed using non-invasively collected samples such as endometrial fluid or peritoneal lavage fluid [16,20]. The mouse xenograft model is useful for studying the mechanism of tumor progression and the effect of drugs in vivo [23]. The exploration of biomarkers using an in vitro model and database analysis has made great progress. With advancement, it is necessary to develop a bioimaging system that enables precise inspection [23].

## 4. Conclusions

**Figure 2:**
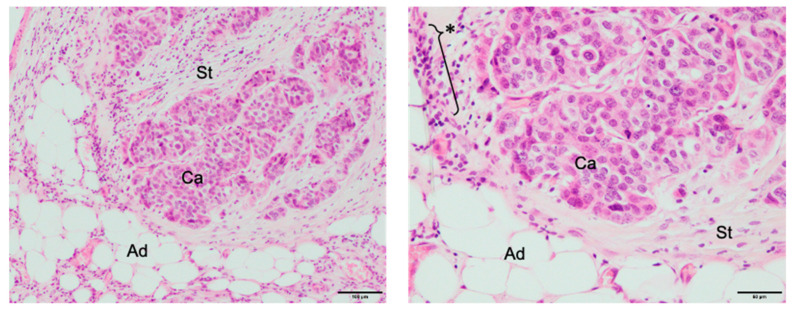
Breast cancer microenvironment. Ad, adipocytes; Ca, carcinoma cells; St, stroma area; *, aggregation of inflammatory cells. Left: low magnification (Scale bar, 100 μm); Right: high magnification of Left (Scale bar, 50 μm).

Cancer tissues contain parenchymal carcinoma cells and stromal cells. The intratumoral stroma is composed of fibroblasts, adipocytes, inflammatory cells, and blood capillaries including pericytes and endothelial cells [24] (Figure 2).

It has been noted that the cancer microenvironment defines the properties of cancer. Additionally, this Special Issue also reports on the search for biomarkers and prognostic factors that focus on the cancer microenvironment in breast and endometrial cancers. Recently, the significance of other hormone receptors such as AR has been clarified in endometrial and breast cancers [25,26,27]; however, various hormone-dependent mechanisms should be elucidated for new therapeutic strategies for both cancers. Database analysis and in vitro research are the most important methodologies for exploring various markers for cancer research. Furthermore, pathological analysis considering the cancer microenvironment and verification using the latest analysis using a mouse xenograft model will lead to the success of translational research.
